# Cardiovascular disease, self-care and emotional regulation processes in adult patients: balancing unmet needs and quality of life

**DOI:** 10.1186/s13030-022-00249-y

**Published:** 2022-09-30

**Authors:** Eleonora Cilli, Jessica Ranieri, Federica Guerra, Claudio Ferri, Dina Di Giacomo

**Affiliations:** 1 Laboratory of Clinical Psychology and Psychoncology, University of L’Aquila, L’Aquila, Italy; 2 Life, Health and Environmental Sciences Department, University of L’Aquila, Via Spennati n. 1, 67010 L’Aquila, Italy; 3 Internal Medicine Division, S. Salvatore Hospital, ASL1 AQ, L’Aquila, Italy

**Keywords:** Cardiovascular disease, Self-care behaviors, Quality of life, Psychological dimensions

## Abstract

**Background:**

Cardiovascular disease is a chronic non-communicable illness that causes more than half of all deaths across Europe. Unhealthy lifestyle, inadequate adherence to medical prescriptions, themselves associated with psycho-emotional disorders are considered risk factors for reduced quality of life as well physical condition.

**Objective:**

Aim of our study was to understand predictive factors for disease management by evaluating psychological aspects, self-care processes and emotional regilati0on in CVD outpatients.

**Methods:**

An observational study was conducted. Sixty-one patients, age 18–75 years (M 56.4 ± sd 12.0), diagnosed with CVD participated in the study. The psychological battery was administered during clinical follow-up oriented to detect emotional and psychological dimensions as well adaptive behavioral and quality of life by standardized questionnaire/scales.

**Results:**

Finding showed that emotional dysregulation might influence QoL, particularly significant effect of awareness (β= 0.022; SE = 1.826; *p* < 0.002), goals (β = - 0.54; SE = 1.48; *p* < 0.001) and clarity (β = - 0.211; SE = 2.087; *p* < 0.003). The results also suggest that the mediated effect accounted for awareness index was 18.7% (*R*^2^ = 0.187) of the variance; goals index 62.8% (*R*^2^ = 0.628) of the variance and, then significant mediated effect of clarity was 58.8% (*R*^2^ = 0.588) of the variance. This evidence suggests that the relationship between triggers and QoL is mediated by emotional dysregulation indexes.

**Conclusion:**

In clinical practice psychological screening can be an effective tool for detecting predictive factors in the management of the CVD patient's health and adherence to medical treatment: the screening of predictive psychological factors for allowing a good clinical condition management and a self-care empowerment aimed at increasing psychological well-being and the Quality of Life by planning adequate integrated and multidisciplinary support.

## Introduction

Cardiovascular disease (CVD) is a chronic physical disease affecting the heart or blood vessels, and it is one of the main causes of death and disability but is can often largely be prevented by leading healthy lifestyle and by efficacy of pharmacological treatments for blood pressure [1]. Clinically, cardiovascular events may occur randomly but are often triggered by physical exertion or emotional stressors and are considered acute risk factors for cardiovascular events [2, 3]. In recent years, several emotional (or mental) triggers, including environmental factors, patient behaviors, and extreme emotional reactions, have been studied for their proximate association to cardiovascular event onset. These triggers—which may in turn cause an extreme emotional disturbance—range from external events of short duration, such as earthquakes or a beloved team’s loss of a football match to acute manifestations of more chronic internal emotional dispositions [4, 5].

According to several studies, hypertension is the main risk factor for the development of stroke, coronary heart disease, insufficiency congestive heart and chronic kidney disease [6]. Moreover, among patients with cardiovascular disease, depression and/or anxiety are very common, with a prevalence ranging from 15 to 50%. Psycho-emotional disorders (in particular, depressive disorders) are associated with increased risk for incident cardiovascular events, rehospitalization, all-cause and cardiovascular mortality both in patients with overt cardiac disease; unhealthy lifestyle, inadequate adherence to medical prescriptions, themselves associated with psycho-emotional disorders are considered risk factors for reduced quality of life as well physical condition [7, 8].

The management of CVD is based on the self-care ability: acquiring specific skills and developing coping strategies to achieve healthy goals, to improve adaptive behaviors, and then to promote the self-efficacy [9]. Therefore, patient empowerment by engagement approach seems to be significant in clinical practice and the individual involvement is precious and fundamental [10]. To date, few studies have been focused on predictive psychological factors of CVD patient long term adherence and well-being. According to a new taxonomy, thought adherence to medications represents a highly prevalent, associated with increased morbidity and mortality, costly to manage, and until recently a very much neglected aspect of therapeutics; moreover, growing literature shows the pervasiveness of poor adherence to appropriately prescribed medications [11]. Non-persistence is one of the most common causes of poor adherence in hypertension with 50% of patients having stopped their treatment at 1 year. Obviously, a lack of persistence has a major influence on CVD control as patients remain off medication for long periods [12, 13].

Aim of our study was to understand the predictive factors of disease management: primary endpoint was to investigate the influence of emotional characteristics, emotion regulation, self-care process, and Quality of Life (QoL) in adult CVD patients; secondary endpoint was to analyze the relationship between psycho-emotional dimensions and cardiovascular disease investigating the ability of patients to manage the own health as well QoL.

## Materials and methods

### Ethic approval

The study was conducted according to the guidelines of the Declaration of Helsinki and approved by the Institutional Review Board of the University of L’Aquila, Italy (Prot. No.37590/2021).

### Participants

Participants of the study have been 61 patients (39F, 22 M) in range age 18–75 years (mean = 56.4 ± SD 12.0) with cardiovascular disease diagnosis (hypertension aware hypertensive (physician-diagnosed hypertension and/or taking antihypertensive medication). Inclusion criteria were as follows: a) > 18 years old; b) diagnosis of cardiovascular disease, c) being outpatients; d) following cardiovascular pharmacological treatment. Exclusion criteria were a) premorbid depression and/or anxiety, b) alcohol or substance abuse; c) no pharmacological treatment; e) no Italian language speaking. We contacted 70 eligible patients, and 61 of them provided informed consent: 2 patients were not interested in participating, 5 of them claimed no time for participations, and then 2 were not appropriate for language (they didn’t speak Italian). The demographic characteristics of the sample are presented in Table 1.

**Table 1:** Demographic characteristics of the sample

	**CVD (*N* = 61)**
**Age (years)**	X 55.4 SD ± 11.32
**Age groups**: n (%)	
≤ 58 years	31 (50.82)
> 58 years	30 (49.18)
**Gender:** n (%)	
Male	22 (36.07)
Female	39 (63.93)
**Marital Status:** n (%)	
Single	17 (27.87)
Married	44 (72.13)
**Educational level**: n (%)	
Graduate	14 (22.95)
No graduate	47 (77.05)
**Occupational status**: n (%)	
Unemployed	31 (50.82)
Employed	30 (49.18)
**Trigger** n(%)	
Mental trigger (MT)	33/(54)
Physical trigger (PT)	28(48)

*Note*: *CVD* Cardiovascular diseases

### Procedure

Medical staff in the Clinical Medicine Division (Director Prof. C. Ferri) identified eligible patients, who have been enrolled during follow-up by medical protocol for management of pharmacological treatment. Written informed consent was obtained at the time of enrolment. The clinical data were collected from medical files. Trained study staff performed a standardized physical examination on participants and administered a structured questionnaire. Participants were asked dichotomous questions, “Were you engaged in heavy physical exertion?” and “Were you angry or emotionally upset?”.

Trained clinical psychologists (blinded to the objectives of the study) conducted the psychological evaluations in a quiet, dedicated room. The evaluations lasted 20 min. Participants completed the measures during their scheduled follow-up. Data were collected anonymously.

## Measures

### Sociodemographic variables

Two types of participant information were collected. First, demographic, and clinical variables were collected by medical records. Demographic data were marital status, having children, being employed, education. Clinical data were related to the triggers, disease severity, pharmacological treatment. Second, psychological measurements have been conducted by digital testing by touch screen technological solution (tablet).

### Psychological measurement

Psychological battery was composed of standardized tests measuring emotional traits (depression, anxiety, stress), Quality of Life, Self-care, and Emotion Regulation variables. Psychological assessment was conducted during scheduled medical follow-up. All tests were applied by Italian population adaptation.

#### Depression Anxiety Stress Scales 21 (DASS-21) [14]

The DASS-21 is a test that measures the degree of severity of n.3 emotional indexes: depression, anxiety, and stress. It is composed of 21 items based on four-point Likert-type scale.

#### Self-Care of Chronic Illness Inventory(SC-CII) [15]

The SC-CII is a 20-item questionnaire to assess the self-care competence in chronic disease. the self-care is the decision-making process involving illness management related to the health promoting; the test is featured by 4 indexes: a) self-care maintenance, b) self-care monitoring, c) self-care management, and self-efficacy. Self-care maintenance reflects primarily health promoting and maintenance behaviours such as exercise and taking medication as prescribed. Self-care monitoring involves checking oneself for changes in signs and symptoms. Self-care management reflects the response to changes in signs or symptoms when and if they occur (e.g. adjusting diet or medication based on detection and interpretation of symptoms). The scoring is based on sub-scale for each self-care indexes (cut-off is 70).

#### World Health Organization Quality Of Life – Brief (WHOQOL) [16]

The WHOQOL is a test to measure the self-perception in life, in the context of the culture, and value systems in which the patient lives and in relation to own goals, expectations, standards and concerns; it is a 26-item test composed of n. 4 domains: 1) physical health (includes items on mobility, daily activities, functional capacity, energy, pain, and sleep), 2) psychological health (includes items on self-image, negative thoughts, positive attitudes, self-esteem, mentality, learning ability, memory concentration, religion, and the mental status), 3) social relationships (contains questions on personal relationships, social support, and sex life), 4) and environmental health (that covers issues related to financial resources, safety, health and social services, living physical environment, opportunities to acquire new skills and knowledge, recreation, general environment (noise, air pollution, etc.), and transportation. WHOQOL-BREF also includes QOL and general health items. Each item is scored from 1 to 5 on a response five-point Likert scale.

#### Difficulties in Emotion Regulation Scale 20 (DERS) [17]

The DERS is a test to assess individual differences in the ability to identify, accept and manage emotional experiences; the test is composed of 6 indexes: a) Non acceptance, b) Goals, c) Impulse, d) Awareness, e) Strategies, f) Clarity.

### Statistical analyses

We conducted the observational study to evaluate emotional characteristics, emotion regulation, self-care process, and Quality of Life (QoL) in young CVD patients.

All data were carefully double-checked for possible miscoding, distribution of values, and updating of missing values. Continuity variables were described using median and DS (Medians, Standard Deviations). Categorical variables are described in percentages. Participants were divided into groups based on acute triggers: mental trigger and physical trigger. Descriptive statistics were conducted to analyze the emotional dimensions, behavioral characteristics as well QoL. Comparison of difference between two groups of continuous variables using Person’s correlations; t-Test statistical analysis and linear regression were applied to detect the relation across the psychological variables. Then, general mediation analysis was conducted to verify the effect of psychological dimensions on QoL of patients. In this study, all analyses were performed by Jamovi stat software. The level of significance adopted was α < 0.05.

## Results

Descriptive analyses were conducted on emotional and behavioral data: Table 2 reported the mean values (and standard deviations) of the participants in psychological testing.

**Table 2:** Psychological characteristics of participants

	**Mean**	**SD**
WHOQOL		
Physical	98.8	21.6
Psychological	86.6	16.9
Social	44.4	10.7
Environment	117.3	21.4
DASS-21		
Depression	9.4	9.5
Anxiety	9.6	9.7
Stress	15.3	10.4
DERS		
Non Acceptance	10.5	5.5
Awareness	9.5	3.0
Goals	9.0	3.8
Clarity	5.4	2.7
Impulse	7.0	3.3
SELF CARE		
Maintenance	65.3	18.2
Monitoring	61.9	17.3
Management	58.4	21.3
Self Efficacy	39.2	7.3

### Acute triggers: emotional and behavioral outcome

First, we wanted to analyze the relationship between acute triggers and emotional outcome; the participants have been distributed into 2 groups by acute triggers: mental trigger (MT) and physical trigger (PT): in Table 1 the distribution was reported. t-Test statistical analysis was performed comparing the acute triggers groups and each behavioral (WHOQOL) and emotional (DERS, DASS-21, SELF-CARE) data.

**Fig. 1:**
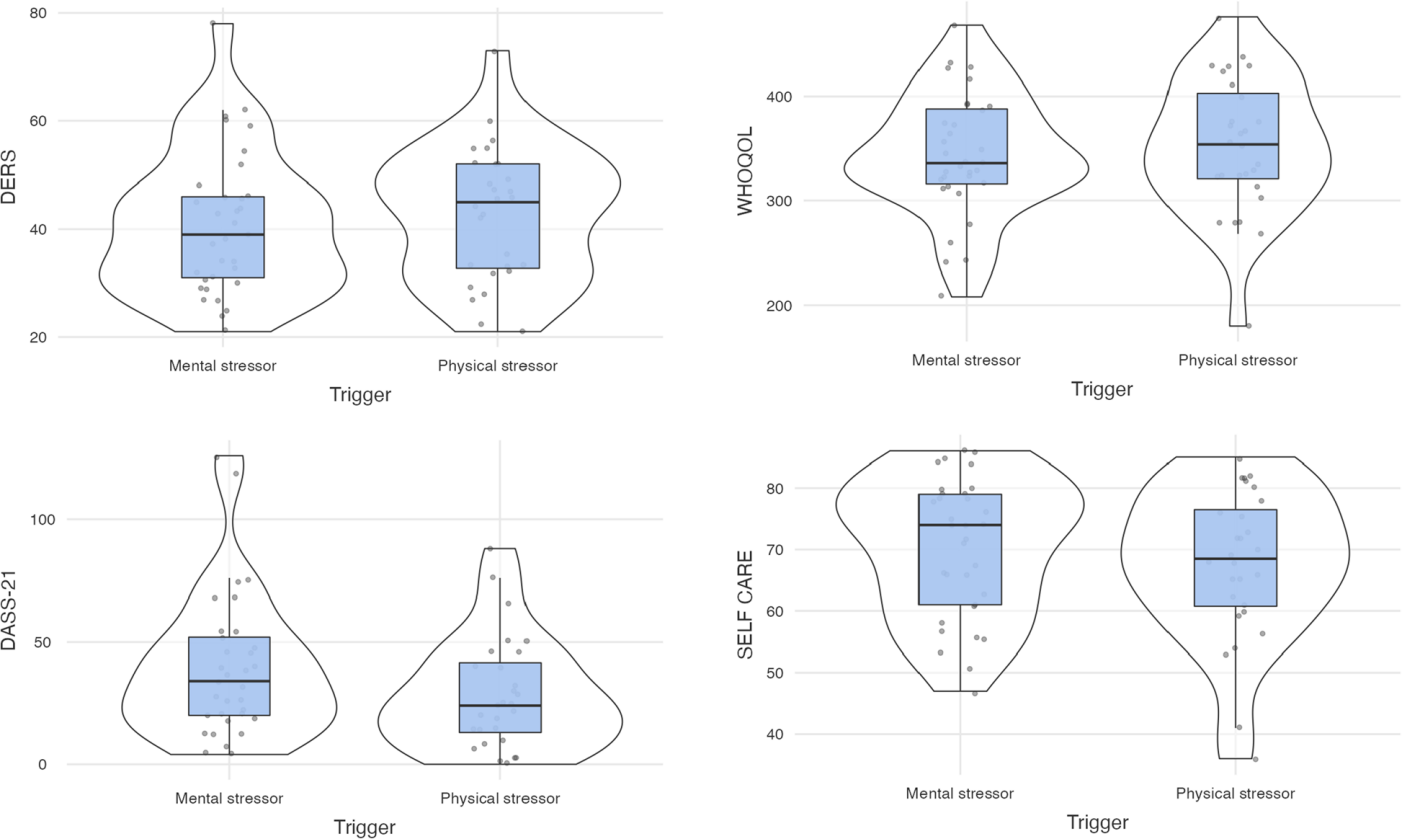
Representation of emotional traits and behavioral dimensions for CVD patients

Table 3 shows the results regarding the comparison between acute trigger groups (MT and PT) on each domain of QoL (physical, psychological, social and environment) detected by WHOQOL test. The results showed no significant differences between participants diagnosed with physical and emotional regarding QoL scores. Table 4 shows the results regarding the comparison between trigger groups on each emotional dimensions (depression, anxiety, stress) detected by DASS-21 test. The result showed no significant difference. Table 5 shows the results regarding the comparison between trigger groups on each behavioral indexes (non-acceptance, awareness, goals, clarity, impulse) detected by DERS test. The result showed no significant difference. Table 6 shows the results regarding the comparison between trigger groups on each self-care ability (maintenance, monitoring, management, self-efficacy) detected by SELF-CARE test. The result showed no significant difference. In Fig. 1 were reported the graphical representations of descriptive analyses emotional and behavioral dimensions. Considering the acute triggers, mental or physical factor on CVD onset didn’t seem influence the psychological dimensions as well self-care behaviors.

**Table 3:** Comparison of participants with mental and physical acute triggers on WHOQOL score by t-Test statistical analysis

**QoL**	**Physical Trigger**		**Mental Trigger**		t	p	Cohen’s d
	M	SD	M	SD			
Physical	100.5	29.3	97.4	19.8	-0.55	0.58	-0.14
Psychological	86.2	17.0	86.9	17.0	0.14	0.88	0.03
Social	46.0	9.8	43.1	11.3	-1.03	0.30	-0.26
Environment	119.2	22.2	115.7	20.9	-0.63	0.52	-0.16

*QoL* Quality of Life

**Table 4:** Comparison of participants with mental and physical acute triggers on DASS-21 score by t-Test statistical analysis

**DASS-21**	**Physical Trigger**		**Mental Trigger**		t	p	Cohen’s d
	M	SD	M	SD			
Depression	8.5	7.8	10.1	10.8	0.68	0.49	0.17
Anxiety	7.0	8.1	11.8	10.5	1.94	0.05	0.49
Stress	12.9	10.1	17.3	10.4	1.68	0.09	0.43

**Table 5:** Comparison of participants with mental and physical acute triggers on DERS score by t-Test statistical analysis

**DERS**	**Physical Trigger**		**Mental Trigger**		t	p	Cohen’s d
	M	SD	M	SD			
Non Acceptance	11.3	5.9	9.8	5.1	-1.06	0.29	-0.27
Awareness	9.2	3.1	9.8	3.0	0.79	0.42	0.20
Goals	9.6	3.8	8.5	3.7	-1.18	0.23	-0.30
Clarity	5.7	2.7	8.5	3.7	-0.67	0.50	-0.17
Impulse	6.6	2.5	7.4	3.9	0.93	0.35	0.23

**Table 6:** Comparison of participants with mental and physical acute triggers on SELF CARE score by t-Test statistical analysis

**SELF CARE**	**Physical Trigger**		**Mental Trigger**		t	p	Cohen’s d
	M	SD	M	SD			
Maintenance	64.2	18.5	66.2	18.3	0.42	0.67	0.10
Monitoring	61.1	16.5	62.2	18.2	0.33	0.73	0.08
Management	54.7	22.6	39.5	7.5	1.28	0.20	0.32
Self efficacy	38.9	7.1	39.5	7.5	0.29	0.77	0.07

### Psychological dimensions featuring cardiovascular disease

Suddenly, we wanted to analyze correlation between examined psychological dimensions. Person’s test evidenced the positive effect between emotional regulation (DERS) and psychological distress (DASS-21) dimensions (*r* = 0.59, *p* = 0.001), whereas negative effects emerged between emotional regulation and QoL (WHOQOL) (*r* = -0.71, *p* = 0.001), and then QoL and psychological distress (*r* = -0.52, *p* = 0.001) (see Tables 3 and Table 7).

**Table 7:** Person correlation on psychological dimensions

	**SELF CARE**	**DERS**		**WHOQOL**		**DASS-21**	
SELF CARE							
Pearson's r	—						
*p*-value	—						
DERS							
Pearson's r	-0.102	—					
*p*-value	0.4336	—					
WHOQOL							
Pearson's r	0.063	-0.710	***	—			
*p*-value	0.6300	< .0001		—			
DASS-21							
Pearson's r	-0.044	0.590	***	-0.521	***	—	
*p*-value	0.7355	< .0001		< .0001		—	

*Note*. * *p* < .05, ** *p* < .01, *** *p* < .001

Then, we wanted to verify the predictive effect of psychological distress and emotional dysregulation on Quality of Life: the hierarchical linear regression was performed with QoL dependent variable, controlling for emotional and physical triggers and emotional dysregulation and psychological distress as covariates. Triggers was added as a controlling variable at the first block; the total score of DERS at the second block; the total score of DASS-21 at the third block. Statistical analyses showed the predictive effect of emotional dysregulation (*R*^2^ = 0.52; *p* < 0.001); no difference on triggers. The results were reported in Table 8.

**Table 8:** Linear regression with QoL as dependent variable

Model Fit Measures							
**Model**			**R**		**R**^**2**^		
1			0.079		0.006		
2			0.721		0.520		
3			0.727		0.528		
Model Comparisons							
**Comparison**							
**Model**		**Model**	**ΔR**^**2**^	**F**	**df1**	**df2**	***p***
1	-	2	0.514	62.037	1	58	< .0001
2	-	3	0.009	1.032	1	57	0.3140
Model Coefficients—WHOQOL							
**Predictor**				**Estimate**	**SE**	**t**	***p***
Trigger:							
Physical stressor – Mental stressor				12.726	12.430	1.024	0.3103
DERS				-3.297	0.592	-5.567	< .0001
DASS-21				-0.292	0.288	-1.016	0.3140

### Impact of emotional dysregulation in QoL

**Fig. 2:**
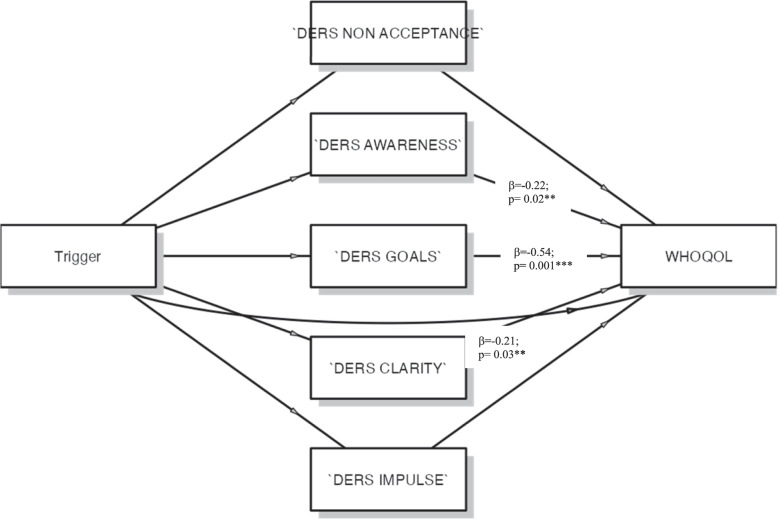
Diagram model of mediation analisys

Then, to assess the hypothesis that emotional dysregulation might influence QoL through acute triggers, mediation analysis was performed. Figure 2 report the diagram model of statistical analysis. The outcome variable was QoL, and the mediator was emotional dysregulation indexes. To take the impact of trigger into account, it was added as independent variable in the model. The results showed the effect of some emotional dysregulation of QoL: significant effect of awareness index on (β = 0.022; SE = 1.826; *p* < 0.002; CI = -7.79– -0.63); goals index was significant on QoL (β= -0.54; SE = 1.48; *p* < 0.001; CI = -5.21– -0.54); significant effect of clarity on QoL (β= -0.211; SE = 2.087; *p* < 0.003; CI = -8.58– -0.41). The results also suggest that the mediated effect accounted for awareness index was 18.7% (*R*^2^ = 0.187) of the variance; goals index 62.8% (*R*^2^ = 0.628) of the variance and, then significant mediated effect of clarity was 58.8% (*R*^2^ = 0.588) of the variance. This evidence suggests that the relationship between triggers and QoL is mediated by emotional dysregulation indexes.

## Discussion

Aim of the study to examine the relationship among individual psychological dimensions, QoL, self-care process of outpatients towards to CVD health management.

Finding evidenced the impact of cardiovascular disease on QoL by emotional regulation processes and emotional dimensions. No significant direct effect of acute triggers on emotional as well behavioral aspects: finding highlighted the good ability of CVD patients in the psychological adaptation to the own health condition; the emotional regulation process resulted impact positively Quality of Life as well as well-being. In the scenario of onset of cardiovascular disease, acute triggers didn’t seem to be relevant, whereas the emotional regulation process resulted to determine QoL as well psychological adaptation: awareness, goals and clarity indexes significantly impacted CVD patient outcome being related causal pathway: those psychological dimensions can mediate the QoL.

Depression, anxiety, and stress didn’t appear significantly relevant by acute triggers: CVD patients could suffer some psychological negative emotion but, in our research, cannot be related to the onset of disease.

Several studies performed the emotional vulnerability of CDV patients by the incidence of psychopathological outcome that could affect the QoL and reduce the well-being [18, 19]. An interesting review highlighted the high incidence of psycho-emotional issues as predictive factors for risk for recurrence of cardiovascular events as well rehospitalization [7]. Secondary effect could be the unexpected pharmacological treatment outcomes as well the exacerbated vulnerability of older CVD patients reducing adherence to the prescribed medical therapy [20]. Considering mentioned side-effects towards to poor adherence, QoL and health management may have serious adverse effects in terms of morbidity and mortality: in particular, non-persistence is one of the most common causes of poor adherence in CVD patients [11]. In line with this perspective, our findings showed negative correlations between emotional regulation and patient ability to monitor and recognize early signs and symptoms of CVD and, then self-efficacy in carrying out specific self-care behaviors as well QoL. Psychological dimensions seem have an important role into CVD disease management from patient side: low awareness about own mental and physical conditions, no well-defined disease oriented-goals and, lack of clarity about own feelings emerged significant factors for QoL mediating (positively/negatively) the CVD physical and health management. Medical actions and health management strategies might involve the individual into complex process of patient engagement.

Clinical follow up, as well biomarkers exams need to be integrated by psychological dimensions into biopsychosocial approach for efficient health management: joining mental and physical treatments, CVD patients might improve their adherence and related health outcome. Tailored awareness, well-progressively defined goals (step by step), and then fitted emotions health feelings could be key points into medical-patient relationship to reinforce and improve the QoL and the persistent behaviors, basic for adherence medications and finally to boost the coping strategies for effective compliance.

Further our finding was the identification of similar impact of acute trigger. Physical and mental triggers didn’t impact differently the self-care ability and emotional regulation: CVD patients seemed overcome the onset of disease being focused on the pathology. Some studies highlighted the relevance of mental triggers in vulnerable individuals, referring to stress, anxiety and depression as mechanisms for acute triggers in CVD onset [4, 21] but the topic is controversial: according to opposite point of view, the absolute impact of acute emotional triggers will be greater among individuals with an elevated baseline CVD risk, such that the effect of two episodes of anger daily would vary from approximately one excess cardiovascular event per 1000 person-years in a low-risk population [5].

Certainly, vulnerable individual could reflect multifactorial clinical symptoms and comorbidity for psychopathological signs ongoing; anyway, present study didn’t confirm the prodromal aspects of trigger each other.

Our study also has several limitations. First, it includes a small sample size that may not be representative and may limit the power to detect significant differences. Second,. due to the relatively small sample size, a sex/gender-based analysis was not feasible, so it is unknown whether the items perform differently in men versus women. Third, although we controlled for several sociodemographic, lifestyle and health status variables, residual confounding cannot be ruled out.

In conclusion, in clinical practice psychological screening can be an effective tool for detecting predictive factors in the management of the CVD patient's health and adherence to medical treatment: the screening of predictive psychological factors for allowing a good clinical condition management and a self-care empowerment aimed at increasing psychological well-being and the Quality of Life by planning adequate integrated and multidisciplinary support. The identification and appropriate correction of these factors might contribute positively to clinical benefits: for instance, poor adherence to CVD pharmacological therapy could be addressed by identifying the emotional characteristics, subjective emotion ability, and self-care process of patients who are or may be at risk of non-persistence.

## Data Availability

Obtained data and materials were based on information about the journal and are available only to editorial board members.

## Abbreviations

CVD: Cardiovascular Disease
QoL: Quality of Life
DASS-21: Depression Anxiety Stress Scales 21
SC-CII: Self-Care of Chronic Illness Inventory
WHOQOL: World health organization quality of life – brief
DERS: Difficulties in Emotion Regulation scale 20
